# ENDOCOR: a nationwide consortium of endocarditis teams—initiating a registry for infective endocarditis within the Netherlands Heart Registration

**DOI:** 10.1007/s12471-025-01979-8

**Published:** 2025-09-03

**Authors:** Annelot J. L. Peijster, Floris J. Heinen, Sabrine Douiyeb, Michelle D. van der Stoel, Jasper L. Selder, Anouk G. W. Watson-de Lepper, Patrick Houthuizen, Matthijs F. L. Meijs, Linda M. Kampschreur, Simon Schalla, Bhanu N. M. Sinha, Annelies L. M. Bakker, Ilse J. E. Kouijzer, Francisca Nijland, C. H. Edwin Boel, Robert K. Riezebos, Mariëlle G. J. Duffels, Jesper Hjortnaes, Robert J. M. Klautz, Nelianne J. Verkaik, Marco C. Post, Steven A. J. Chamuleau, Otto Kamp, Wilco Tanis

**Affiliations:** 1 Amsterdam University Medical Centre, Amsterdam, The Netherlands; 2 Haga Teaching Hospital, The Hague, The Netherlands; 3 Netherlands Heart Registration, Utrecht, The Netherlands; 4 Catharina Hospital, Eindhoven, The Netherlands; 5 Medical Spectrum Twente, Enschede, The Netherlands; 6 Medical Centre Leeuwarden, Leeuwarden, The Netherlands; 7 Maastricht University Medical Centre, Maastricht, The Netherlands; 8 University Medical Centre Groningen, Groningen, The Netherlands; 9 Amphia Hospital, Breda, The Netherlands; 10 Radboud University Medical Centre, Nijmegen, The Netherlands; 11 Our Lady’s Hospital, Amsterdam, The Netherlands; 12 University Medical Centre Utrecht, Utrecht, The Netherlands; 13 Isala Zwolle, Zwolle, The Netherlands; 14 North West Hospitals, Alkmaar, The Netherlands; 15 Leiden University Medical Centre, Leiden, The Netherlands; 16 Erasmus Medical Centre, Rotterdam, The Netherlands; 17 St. Antonius Hospital, Nieuwegein, The Netherlands

**Keywords:** Infective endocarditis, Endocor, Registry, Gaps in evidence, Clinical equipoise, First national data

## Abstract

***Background*:**

Despite advancements in diagnostics and treatment strategies, infective endocarditis continues to carry a substantial morbidity and mortality risk. In addition, the field of infective endocarditis contains many gaps in evidence, as international guidelines are predominantly based on low-level evidence. To improve infective endocarditis care and survival rates in the Netherlands, adequate evaluation of diagnostics, treatment strategies and outcomes is essential.

***Methods*:**

To address this need, a new infective endocarditis registry has been developed by the multidisciplinary ENDOCOR working group with the aim of facilitating nationwide quality control, improving infective endocarditis patient care, and contributing to the numerous gaps in evidence. To optimize data collection, facilitated by the Netherlands Heart Registration (NHR), a pilot project was launched in January 2023 across three selected hospitals.

***Results*:**

The findings from the first 150 registered patients were presented to highlight the registry’s potential. Following the pilot, many more centres have initiated data collection, demonstrating national engagement and scalability of the initiative.

***Conclusion*:**

This article outlines the purpose of ENDOCOR, presents initial pilot data and illustrates the potential of the new national infective endocarditis registry to enhance patient care and support future research.

## Introduction

Infective endocarditis (IE), characterized by its heterogeneity and severity, carries a substantial morbidity and mortality risk [1–5]. This primarily stems from the risk of severe complications in IE patients, such as sepsis, embolisms, heart failure and local peri-annular extension of the infection. In addition, the disease is challenging to diagnose early, as its primary symptoms, such as fever and chills, are common and nonspecific [4, 5]. International guidelines, including diagnostic criteria, are continually being refined; however, the overall level of evidence in the field of IE remains remarkably low. The latest European Society of Cardiology (ESC) guideline for the management of IE was released in October 2023 and primarily relies on level C evidence, thus highlighting the numerous gaps in evidence [5]. For example, there is a new class 1, yet level C recommendation for surgery in patients with early prosthetic valve endocarditis (PVE), despite the lack of supporting evidence, as well as instances of clinical equipoise, such as the ongoing search for the optimal timing for surgery in IE patients [1, 3, 5, 6]. To enhance IE care, large-scale, prospective multicenter studies are essential. In response, the ENDOCOR working group has established a new IE registry in the Netherlands to help bridge the existing gaps in evidence.

## Background and rationale

### *The epidemiological profile of IE and the 2023 ESC guideline*

Although the disease is rare (around 10 cases per 100,000 person years), IE has exhibited not only a consistently high mortality, but also a noticeable increase in incidence over the past three decades, seemingly reaching a new plateau according to recent data [1, 3, 7–9]. This apparent increase in incidence is considered to be of multifactorial origin, including the aging of the population, more opportunities for health care associated IE (which includes more frequent staphylococcal IE) due to the wider range of available treatment options hospital-wide and an increasing frequency of prosthetic heart valve implantation, including transcatheter valve implantation, as well as cardiac electronic device implantation. Consequently, the profile of typical causative agents for IE has shifted [1, 3–5]. Another important factor contributing to the increasing incidence could be that the disease is diagnosed more often due to improved awareness, diagnostic criteria and multiple imaging options [5].

**Fig. 1:**
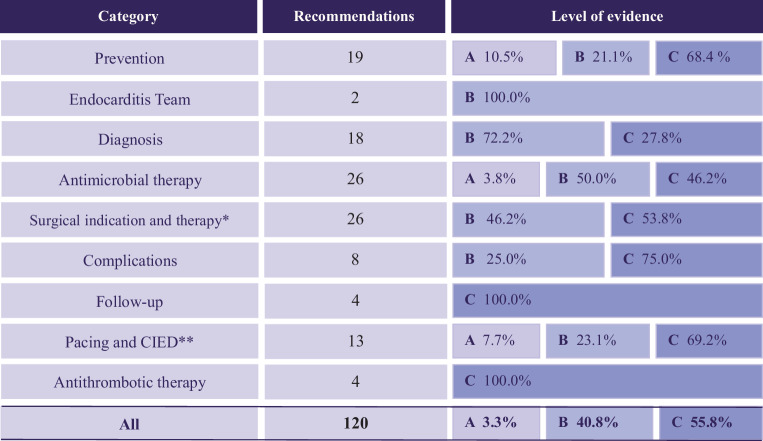
Levels of evidence in the ESC guideline of the management of IE. *ESC* European Society of Cardiology, *IE* infective endocarditis. *This category includes surgical indications after neurological complications. **This category includes pacing indication after conduction complications

The 2023 ESC guideline for the management of IE reflects the changing epidemiological profile with some updates in diagnosis and treatment. The new key recommendations focus on prevention, diagnostic criteria, multimodality imaging, oral antibiotic therapy, surgical indications, and timing. In addition, the recommendation for the ‘Endocarditis Team’ was upgraded (to class I, level B), which is expected to be of great value for patient care [5, 10]. Regarding prevention, both the groups eligible for antibiotic prophylaxis and those receiving other preventive measures were expanded [5, 11–18]. For the diagnosis of IE, the major ESC criteria now include a broader range of causative pathogens than before, and the minor ESC criteria now include hematogenous osteoartricular spread [5, 19]. Furthermore, the new guideline emphasizes more frequent use of transesophageal echocardiography (TOE), even in conjunction with positive transthoracic echocardiography (TTE), along with the reinforcement of cardiac computed tomography (CT) angiography, 18F-Fluorodeoxyglucose positron-emission tomography/computed tomography (FDG-PET/CT), and other extracardiac imaging indications such as cerebral magnetic resonance imaging (MRI) [5, 20–24]. A further significant change involves the (class IIa, level A) recommendation for a specific subset of IE patients, allowing the option of oral antibiotic therapy at home for clinically stable individuals, per the findings of the Partial Oral Treatment of Endocarditis trial [5, 25]. Finally, surgery is recommended more frequently, and urgent surgery is performed with quicker timing [5, 26, 27]. Although many improvements in the guideline are considered valuable, they remain predominantly supported by low-level evidence. This is evident across all chapters, with most recommendations based on level C evidence (55.8%), followed by level B (40.8%), of which the majority are derived from observational studies rather than single randomized controlled trials (RCT’s). Notably, only 3.3% of recommendations are supported by level A evidence (Fig. 1), thus highlighting the need for further research to strengthen its clinical foundation [5].

## Methods

### ENDOCOR working group and Dutch national IE registry

**Fig. 2:**
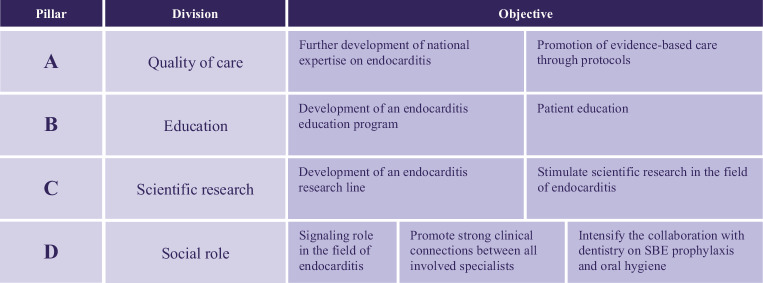
Pillars of the IE working group ENDOCOR. *IE* infective endocarditis, *SBE* subacute bacterial endocarditis

In order to improve IE care, reduce mortality rates, and potentially contribute to the gaps in evidence, adequate evaluation of diagnostics, treatment strategies, and outcomes was needed. In addition, nationwide collaboration was essential for achieving and advancing research collaborations on a larger scale. Therefore, in 2022, the working group for IE in the Netherlands ENDOCOR (analogous to CONCOR, the national registry and DNA-bank of patients with congenital heart disease [28]) was founded to promote and facilitate quality of care, education, and scientific research within the field of IE (Fig. 2). Members of the ENDOCOR group are all specialists with direct involvement in the diagnostic and therapeutic process for IE patients, who participate in the Endocarditis Team.

To achieve the objectives of ENDOCOR, a nationwide registry was initiated in January 2023, in collaboration with the existing quality control infrastructure of the Netherlands Heart Registration (NHR). The NHR enables the use of real-world data to monitor and further improve the quality of care for cardiovascular diseases. In the currently existing registries of the NHR, data on all cardiac interventional, electrophysiological, and surgical procedures are collected [29, 30]. Indeed, the database of cardiac surgery procedures provides invaluable data on surgical IE patients. However, notable limitations hinder a more comprehensive assessment of IE. While the existing registry consists of a sizable IE patient cohort, its primary perspective consists of surgical details (e.g., procedure type, post-operative complications) and leaves out key variables for further IE patient analyses. Most importantly, the current registry does not include any conservatively treated IE patients. Given the heterogeneous nature of IE, adopting a multidisciplinary approach becomes imperative. Therefore, capturing data on causative pathogens of IE, antimicrobial therapies, and performed (cardiac) multimodality imaging is crucial for optimal evaluation of IE patients, and this information has not been captured until now.

In January 2023, the IE registry within the NHR (pilot phase) was initiated in three Dutch hospitals, namely: the Haga Teaching Hospital, The Hague, the Amsterdam University Medical Centre, Amsterdam, and the Catharina Hospital in Eindhoven. Easily accessible and shareable data registration lists have been developed for different electronic health records (HiX and Epic included), thus streamlining the launch of IE registration across all participating hospitals. As integral members of ENDOCOR, we expect that every Dutch hospital offering cardiothoracic surgery will actively participate and register patients via the Endocarditis Team, a process already initiated or confirmed in several centres. As a result, we anticipate the inclusion of nearly all known IE patients in the Netherlands, as per the Dutch Working Group on Antibiotic Policy (Stichting Werkgroep AntibioticaBeleid, SWAB), Federation of Medical Specialists (Federatie Medisch Specialisten, FMS) and ESC (class I, level B) guideline recommendations that every patient with IE should be discussed in an Endocarditis Team of a Heart Valve Center at least once [5, 31]. In the Netherlands, this accounts for an estimated inclusion of around 2000 IE patients annually [5, 32]. The new NHR IE registry is intended as a voluntary registration. Its primary objective is to ensure quality control. As such, if significant variations are found through the registry, discussions regarding the different diagnostic and treatment strategies will be facilitated in a safe setting. In addition, this large-scale and long-term prospectively collected data will offer unique insights into epidemiological changes and temporal trends of IE in the Netherlands and contribute to the many gaps in evidence in the field of IE. This is particularly valuable with the ability to link the various NHR registries. A waiver for informed consent for analysis of the data of the NHR registry data was obtained. When defining the catalogue of variables for this registry, all medical specialists that form the Endocarditis Team were involved and aimed for a carefully considered balance between registration burden and registry benefits. Consequently, specific details such as particular imaging findings or antibiotic susceptibility testing were not included. The outcome is a list encompassing broad yet not overly detailed IE relevant topics (Tab. 1), which in practice (experienced during the pilot) are registered within one minute during the Endocarditis Team, as all necessary information is discussed in the meeting. Moreover, in the future, it will be easier to retrospectively gather additional information about any notable subgroup within the IE cohort.

**Table 1:** List of variables new IE registry.

Variable	Values
Previous valve surgery	Yes/No
Previous IE	Yes/No
Congenital heart disease	Yes/No
Serum creatinine level	*Automatically retrieved from EHR*
Dialysis	Yes/No
Referring hospital	*Name of hospital*
Transesophageal echocardiogram	None/Positive for IE/Negative or inconclusive for IE
Cerebral MRI	None/Positive for IE/Negative or inconclusive for IE
FDG-PET/CT	None/Positive for IE/Negative or inconclusive for IE
Cardiac CT angiography	None/Positive for IE/Negative or inconclusive for IE
Causative pathogen IE	*Choose pathogen group*
ESC criteria major (2023)	0/1/2
ESC criteria minor (2023)	0/1/2/3/4/5
IE diagnosis	Possible/Definite
IE location	Aortic/Mitral/Pulmonic/Tricusid/Native/Prosthetic
IE of device	Yes/No
IE of vascular prosthesis	Yes/No
Peri-annular expansion	Yes/No
Severe valve regurgitation due to IE	Yes/No
Heart failure due to IE	Yes/No
Vegetation length	None/< 10 mm/10–15 mm/15–30 mm/> 30 mm
Distal embolization	Yes/No
Indication for invasive intervention	None/Yes and advised/Yes but not advised due to high risk
Advised clinical antibiotics	*Choose advised regimen*
Intended treatment duration of IV antibiotics	None/4 weeks/6 weeks
Outpatient chronic oral suppressive antibiotics advised (after IV treatment)	Yes/No

*IE* infective endocarditis, *EHR* electronic health record, *MRI* magnetic resonance imaging, *FDG-PET/CT* 18F-Fluorodeoxyglucose positron-emission tomography/computed tomography, *CT* computed tomography, *ESC* European Society of Cardiology, *IV* intravenous

## Results

### *Initial findings from the pilot phase*

To provide a preliminary insight into the new registry, we present herein data from the first 150 IE patients registered in 2023. All patients discussed in the Endocarditis Team with a possible or definite IE (*n* = 72 and *n* = 78, respectively) were included. The median age of patients at diagnosis was 69.5 years (IQR 13), with 70% being male. Prior IE was noted in 13.3% of patients, and 35.3% were referred from other hospitals (Tab. 2).

**Table 2:** First results pilot phase new IE registry (01/2023 to 08/2023).

Variable			Total *n* = 150	Possible IE *n* = 72	Definite IE *n* = 78
Baseline characteristics					
	Age, years, median (IQR)		69.5 (13)	70.5 (14.5)	68.5 (11.8)
	Male sex, *n* (%)		105 (70.0)	46 (63.9)	59 (75.6)
	Previous valve surgery, *n* (%)		46 (30.7)	17 (23.6)	29 (37.2)
	Previous IE, *n* (%)		20 (13.3)	9 (12.5)	11 (14.1)
	Congenital heart disease, *n* (%)		11 (7.3)	3 (4.2)	8 (10.3)
	Dialysis, *n* (%)		6 (4.0)	4 (5.6)	2 (2.6)
Outcomes					
*Hospital*					
	Referred, *n* (%)		53 (35.3)	14 (19.4)	39 (50.0)
*Imaging*					
	Transesophageal echocardiogram				
		Positive for IE, *n* (%)	51 (34.0)	6 (8.3)	45 (57.7)
		Negative/inconclusive for IE, *n* (%)	28 (18.7)	18 (25.0)	10 (12.8)
		None, *n* (%)	71 (47.3)	48 (66.7)	23 (29.5)
	Cerebral MRI				
		Positive for IE, *n* (%)	6 (4.0)	1 (1.4)	5 (6.4)
		Negative/inconclusive for IE, *n* (%)	3 (2.0)	1 (1.4)	2 (2.6)
		None, *n* (%)	141 (94.0)	70 (97.2)	71 (91.0)
	FDG-PET/CT				
		Positive for IE, *n* (%)	34 (22.7)	9 (12.5)	25 (32.1)
		Negative/inconclusive for IE, *n* (%)	22 (14.7)	14 (19.4)	8 (10.3)
		None, *n* (%)	94 (62.7)	49 (68.1)	45 (57.7)
	Cardiac CT angiography				
		Positive for IE, *n* (%)	17 (11.5)	1 (1.4)	16 (20.8)
		Negative/inconclusive for IE, *n* (%)	6 (4.1)	3 (4.2)	3 (3.9)
		None, *n* (%)	125 (84.5)	67 (94.4)	58 (75.3)
*Affected valve/prothesis*					
	Aortic native valve (%)		38 (25.9)	6 (8.6)	32 (41.6)
	Mitral native valve, *n* (%)		22 (14.8)	7 (9.7)	15 (19.5)
	Tricuspid native valve, *n* (%)		3 (2.0)	1 (1.4)	2 (2.6)
	Pulmonary native valve, *n* (%)		2 (1.3)	0 (0.0)	2 (2.6)
	Aortic prosthetic valve, *n* (%)		38 (25.9)	12 (17.1)	26 (33.8)
	Mitral prosthetic valve, *n* (%)		5 (3.4)	2 (2.8)	3 (3.9)
	Vascular prosthesis, *n* (%)		8 (5.4)	3 (4.2)	5 (6.4)
	CIED, *n* (%)		13 (9.0)	8 (11.4)	5 (6.7)
	Location uncertain, *n* (%)		36 (28.8)	36 (62.1)	0 (0.0)
*Amount of affected valves*					
	Multiple valves, *n* (%)		41 (28.3)	12 (17.4)	29 (38.2)
*30-day mortality*					
	Alive, *n* (%)		130 (87.3)	61 (85.9)	69 (88.5)

*IQR* interquartile range, *IE* infective endocarditis, *MRI* magnetic resonance imaging, *FDG-PET/CT* 18F-Fluorodeoxyglucose positron-emission tomography/computed tomography, *CT* computed tomography, *CIED* cardiovascular implanted electronic device

TOE was performed in 52.7% of registered patients, with 64.6% of these yielding positive results for IE. FDG-PET/CT and cardiac CT were conducted in 37.4% and 15.3% of patients respectively, with positive findings for IE in 60.7% of FDG-PET/CT and 73.9% of cardiac CT cases. Cerebral MRI was carried out in 6% of patients, showing positive results for IE in 66.7% (Tab. 2).

**Fig. 3:**
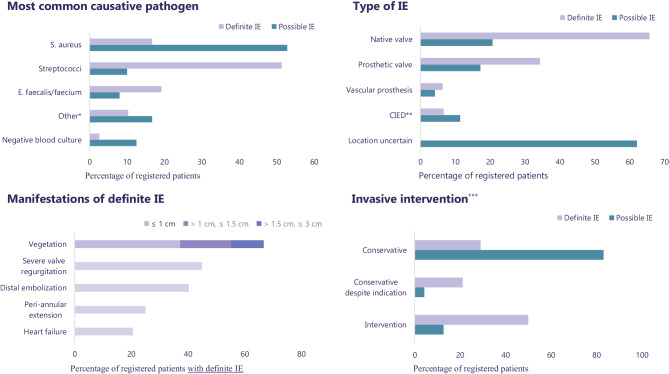
First results pilot phase new IE registry (01/2023 to 08/2023). *IE* infective endocarditis, S. aureus *Staphylococcus aureus*, E. faecalis/faecium *Enterococcus faecalis/faecium*, *CIED* cardiovascular implanted electronic device, *cm* centimeters.* ‘Other’ consists of all causative pathogens besides the ones mentioned above, as well as an unknown result. ** Please note that the CIED group consists of device endocarditis only (pocket infections are not reported here). *** ‘Invasive intervention’ encompasses both CIED extractions and cardiac surgery

Regarding the type of IE, 26% of patients had prosthetic valve endocarditis, and 8.7% were cases of cardiovascular implanted electronic device (CIED) endocarditis. The aortic valve was the most commonly affected valve (both native and prosthetic), and *Staphylococcus aureus* was the most frequently identified causative pathogen in possible IE. Streptococci were the most frequent causative pathogen in patients with definite IE. In the definite IE group, the most common manifestations included severe valve regurgitation, vegetations, and distal embolization (44.9%, 66.7%, and 39.7% respectively). Overall, invasive intervention was indicated in 44.0% of the patients, however intervention was deemed too high-risk for 28.8% of these patients (Fig. 3). Specifically, among patients with definite IE, surgery was indicated in 71.1% of cases and advised in 50.0%. The 30-day mortality rate in this preliminary data was 12.7% (Tab. 2).

## Discussion

### *Potential contributions to clinical equipoise in the field of IE*

While this small cohort from a limited number of (referral) hospitals does not provide sufficient data to fully assess IE care in the Netherlands, it does illustrate part of the registry’s potential. National data will be available to address the many knowledge gaps in the field of IE. In general, specific topics can be explored, such as sex differences in IE, which are highlighted as a key research priority in the ESC guideline, as well as more specific research questions, such as the role of vegetation length and the occurrence of neurological complications, while taking into account the affected valve and causative agent [5]. Other heavily theorized areas of uncertainty could be investigated, such as the use of suppressive antibiotic therapy in inoperable PVE or CIED IE, as well as the potential role of FDG-PET/CT guiding the decisions to start or discontinue antibiotic therapy [33, 34]. Beyond the long-term mortality outcomes, the registry will also monitor factors such as relapse rates, as cases of re-infection will be registered (again), providing important data that may directly influence future decision-making in IE care. In addition, the new registry will provide insights into patients with possible endocarditis, a group that is overlooked in most IE studies. Moreover, the registry will facilitate the evaluation of (new) guideline recommendations and ways to further improve them. For instance, analyzing IE patients switching to oral antibiotic therapy may contribute to the ongoing discussions on whether monotherapy or dual therapy is preferable, or assessing whether surgery should always be considered for patients with early PVE (within 6 months of valve surgery), given that this is now a class I, yet level C, recommendation [5, 35]. Above all, as this registry is part of the NHR, integrating the various registries will enhance the completeness and overall value of the data. One of the well-known clinical equipoises in IE is determining the optimal timing for an indicated surgery. By linking this new registry with the surgical registry, a more extensive and up-to-date dataset will be available to analyze this matter. Similarly, combining the data from the devices registry will provide insights into the widely discussed timing of reimplantation after CIED IE and could help determine the most suitable device for this patient group as well. Finally, it is important to recognize that registry-based clinical trials are becoming increasingly feasible, as they are relatively efficient, cost-effective, and real-world applicable while allowing for long-term follow-up [36, 37]. Although they are not RCTs and require careful consideration of confounding and bias, they provide structured data that can help to fill current evidence gaps and provide better guidance for clinical equipoise.

## Conclusion

A new national IE registry has been developed by the multidisciplinary ENDOCOR working group in collaboration with the NHR. The primary goal is to facilitate nationwide quality control and improve patient care in IE. Given that the field of IE and its guidelines are largely based on low-level evidence, this national registry offers a valuable opportunity to address the existing gaps in evidence.
